# AI-Driven Patient Education in Chronic Kidney Disease: Evaluating Chatbot Responses against Clinical Guidelines

**DOI:** 10.3390/diseases12080185

**Published:** 2024-08-16

**Authors:** Prakrati C. Acharya, Raul Alba, Pajaree Krisanapan, Chirag M. Acharya, Supawadee Suppadungsuk, Eva Csongradi, Michael A. Mao, Iasmina M. Craici, Jing Miao, Charat Thongprayoon, Wisit Cheungpasitporn

**Affiliations:** 1 Division of Nephrology, Texas Tech Health Sciences Center El Paso, El Paso, TX 79905, USA; prakrati.acharya@ttuhsc.edu (P.C.A.); raualba@ttuhsc.edu (R.A.); cacharya@ttuhsc.edu (C.M.A.); 2 Division of Nephrology, Thammasat University Hospital, Pathum Thani 12120, Thailand; pajareek@tu.ac.th; 3 Division of Nephrology and Hypertension, Mayo Clinic, Rochester, MN 55905, USA; supawadee.sup@mahidol.ac.th (S.S.); craici.iasmina@mayo.edu (I.M.C.); miao.jing@mayo.edu (J.M.); thongprayoon.charat@mayo.edu (C.T.); 4 Chakri Naruebodindra Medical Institute, Faculty of Medicine Ramathibodi Hospital, Mahidol University, Samut Prakan 10540, Thailand; 5 Faculty of Medicine, University of Debrecen, 4032 Debrecen, Hungary; csongradi.eva@med.unideb.hu; 6 Division of Nephrology and Hypertension, Department of Medicine, Mayo Clinic, Jacksonville, FL 32224, USA; mao.michael@mayo.edu

**Keywords:** chronic kidney disease (CKD), patient engagement, healthcare–AI collaboration, diseases

## Abstract

Chronic kidney disease (CKD) patients can benefit from personalized education on lifestyle and nutrition management strategies to enhance healthcare outcomes. The potential use of chatbots, introduced in 2022, as a tool for educating CKD patients has been explored. A set of 15 questions on lifestyle modification and nutrition, derived from a thorough review of three specific KDIGO guidelines, were developed and posed in various formats, including original, paraphrased with different adverbs, incomplete sentences, and misspellings. Four versions of AI were used to answer these questions: ChatGPT 3.5 (March and September 2023 versions), ChatGPT 4, and Bard AI. Additionally, 20 questions on lifestyle modification and nutrition were derived from the NKF KDOQI guidelines for nutrition in CKD (2020 Update) and answered by four versions of chatbots. Nephrologists reviewed all answers for accuracy. ChatGPT 3.5 produced largely accurate responses across the different question complexities, with occasional misleading statements from the March version. The September 2023 version frequently cited its last update as September 2021 and did not provide specific references, while the November 2023 version did not provide any misleading information. ChatGPT 4 presented answers similar to 3.5 but with improved reference citations, though not always directly relevant. Bard AI, while largely accurate with pictorial representation at times, occasionally produced misleading statements and had inconsistent reference quality, although an improvement was noted over time. Bing AI from November 2023 had short answers without detailed elaboration and sometimes just answered “YES”. Chatbots demonstrate potential as personalized educational tools for CKD that utilize layman’s terms, deliver timely and rapid responses in multiple languages, and offer a conversational pattern advantageous for patient engagement. Despite improvements observed from March to November 2023, some answers remained potentially misleading. ChatGPT 4 offers some advantages over 3.5, although the differences are limited. Collaboration between healthcare professionals and AI developers is essential to improve healthcare delivery and ensure the safe incorporation of chatbots into patient care.

## 1. Introduction

Chronic kidney disease (CKD) is a serious multisystem disease with increasing prevalence that deleteriously affects an individual’s well-being and mortality [1,2]. Data from the Global Burden of Disease Study reveal that about 10 percent of individuals globally are afflicted with CKD, positioning it at the forefront of present-day medical challenges [3]. The epidemiological evidence highlights the scale of this medical dilemma and emphasizes the pressing need for intervention.

Education plays an essential role in managing diseases [4]. For a multifarious condition like CKD, modifications in an individual’s lifestyle and diet play a crucial role in dictating the course of the disease and its broader health implications [5]. These modifications require not merely the acquisition of knowledge but an intricate comprehension and unwavering commitment in order to apply and alter the individual’s disease course. Personalized educational interventions have, therefore, become foundational in the approach to CKD care. Consistent research findings advocate that when patients are armed with tailored information, their compliance with medical recommendations increases, leading to optimized health outcomes [6].

In the era of digital advancement, traditional patient education methods remain the cornerstone for medical care, but the rise of artificial intelligence (AI) ushers in transformative possibilities [7]. Chatbot models, like ChatGPT, introduced in late 2022, exemplify the fusion of linguistic sophistication and advanced computation for wide application in society, including the medical field, where it has been leveraged for licensing assessments and curriculum evaluations [8,9]. One emerging focus is the use of chatbots for patient education. These AI-driven chatbots excel at simulating human interactions, promising not just accessible but also engaging and tailored patient medical education [10,11]. This innovation addresses a common challenge: that patients often grapple with a deluge of information and struggle to meaningfully apply it to their own unique circumstances. In complex conditions like CKD, this includes specific dietary advice, exercise recommendations, weight management, and lifestyle adjustments [12,13,14,15,16]. Chatbots thus may play a pivotal role in delivering precise, personalized, timely, and clear information, guiding patients to clarity and empowerment [17].

However, every innovative creation requires a thorough examination of its strengths, flaws, and limitations. Thus, this study was designed with a dual purpose. First, it aimed to conduct a critical assessment of the effectiveness of chatbots (various versions of ChatGPT, Bing AI, and Bard AI) to address essential queries related to the lifestyle and nutritional aspects of CKD management. Second, it sought to analyze each chatbot’s evolving performance trajectories and make comparisons among the different AI models. This study’s results have the potential to enhance the medical field’s understanding of the role of AI-driven tools in patient-centered education for CKD, especially given the current scarcity of data.

In this study, we aimed to evaluate ChatGPT 3.5, ChatGPT 4.0, Bing AI, and Bard AI in order to gauge their accuracy and effectiveness as patient education tools for CKD lifestyle and dietary management.

## 2. Materials and Methods

### 2.1. Question Generation from KDIGO Guidelines

Out of the thirteen available Kidney Disease: Improving Global Outcomes (KDIGO) guidelines, three that provided pertinent information regarding lifestyle modification and nutrition were selected for question generation. The selected guidelines were

- “KDIGO 2022 Clinical Practice Guideline for Diabetes Management in Chronic Kidney Disease” [18].
- “KDIGO 2012 Clinical Practice Guideline for the Evaluation and Management of Chronic Kidney Disease” [19].
- “KDIGO 2013 Clinical Practice Guideline for Lipid Management in Chronic Kidney Disease” [20].

Fifteen questions related to lifestyle modification and nutrition were generated following a comprehensive review of these three key KDIGO guidelines (Supplementary Table S1). Nephrologists generated questions based on commonly asked questions raised by patients encountered during clinical practice.

### 2.2. Question Generation from NKF KDOQI Guidelines

Twenty questions from the National Kidney Foundation-Kidney Disease Outcome Quality Initiative (NKF KDOQI) guidelines for nutrition in CKD (2020 Update) [21] related to lifestyle modification and nutrition were generated (Supplementary Table S2). Nephrologists generated questions based on commonly asked questions raised by patients encountered during clinical practice.

### 2.3. Question Interrogation Process for KDIGO Guideline Questions

**Figure 1:**
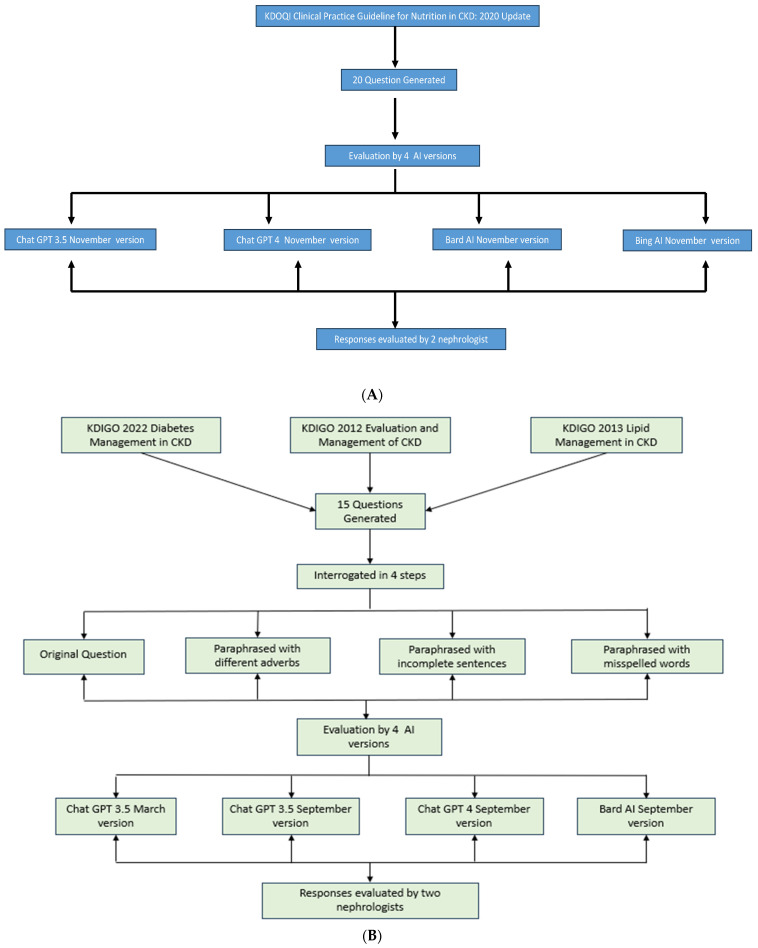
(**A**) Flow diagram depicting interrogation by AI models for KDOQI guideline questions. (**B**). Flow diagram depicting interrogation by AI models for KDIGO guideline questions.

The fifteen questions generated from the selected KDIGO guidelines underwent a rigorous four-step interrogation process to evaluate the versatility and responsiveness of the chatbots, as illustrated in Figure 1A,B. These steps included

- The original question formulation as per the guideline’s recommendations.
- Paraphrasing each question while employing different interrogative adverbs.
- Crafting paraphrased questions with incomplete sentences.
- Formulating paraphrased questions with deliberate misspellings.

Questions generated by the NKF KDOQI guidelines were interrogated using only the original version, as no difference was noted in the answers, even when paraphrasing with different adverbs and using incomplete sentences and misspellings while evaluating questions from KDIGO guidelines.

While our primary goal was to mimic patient queries through paraphrased questions with different adverbs, incomplete sentences, and misspelled words, this approach inadvertently served a dual purpose. By posing these variations within the same timeframe, we effectively examined each question multiple times (the original plus three variations). This method allowed us to assess not only the AI’s ability to interpret diverse question formats but also to evaluate the consistency of responses across these variations.

The questions, generated in the previous step, were subjected to evaluation by distinct AI models, each representing different versions and capabilities:

- ChatGPT 3.5.
- ChatGPT 4.0.
- Bard AI.
- Bing AI.

ChatGPT 3.5 is a generative AI model developed by OpenAI, renowned for its ability to understand context and generate coherent and contextually relevant content. This version is recognized for its capacity to provide responses to a wide range of queries, from straightforward information retrieval to more complex problem-solving tasks. It leverages patterns learned from extensive datasets to generate responses. ChatGPT 4 is an advanced generative AI model also developed by OpenAI. It stands as an evolution of ChatGPT 3.5, with notable improvements in performance, algorithm fine-tuning, and the ability to handle intricate tasks [22].

Bard AI is a specialized generative AI model, developed by Google, that is unique in its proficiency for comprehending and generating narratives. It is built upon the foundation of Google’s Pathway Language Model (PaLM), a cutting-edge technology designed to enhance the model’s narrative generation capabilities. PaLM represents a pathway to a more coherent and contextually aware storytelling, aligning seamlessly with Bard AI’s overarching narrative generation prowess [23]. Bard AI is now referred as Gemini [24].

Bing AI was developed by Microsoft. It was later renamed Microsoft Copilot. It is powered by DALL E2. It was launched in February 2023. Bing also incorporated an image creator within a month.

### 2.4. Evaluative Review by Nephrologist

Two practicing nephrologists independently reviewed the AI-generated responses. Their primary objective was to identify any errors or misinformation that could potentially harm patient care. The evaluation criteria included

(a) Clinical accuracy: Assessing whether the information aligns with current medical knowledge and practice.
(b) Potential for patient harm: Identifying any advice that could lead to adverse outcomes if followed.
(c) Clarity and relevance: Evaluating whether the response directly addresses the question in a clear manner.
(d) Consistency with guidelines: While not the sole criterion, responses were checked against relevant guidelines when significant discrepancies were noted.

### 2.5. Readability Analysis

To assess the readability of both the questions posed and the answers provided by the AI chatbots (ChatGPT 3.5, ChatGPT 4.0, Bing AI, and Bard AI), we employed the Flesch–Kincaid Grade Level formula. This widely used tool in health communication research provides an estimate of the U.S. grade level required to understand the text. We applied this measure to all questions and AI-generated responses to evaluate their accessibility to individuals with varying educational backgrounds.

## 3. Results

### 3.1. ChatGPT 3.5 (March 2023 Version) for KDIGO Guideline Questions

**Figure 2:**
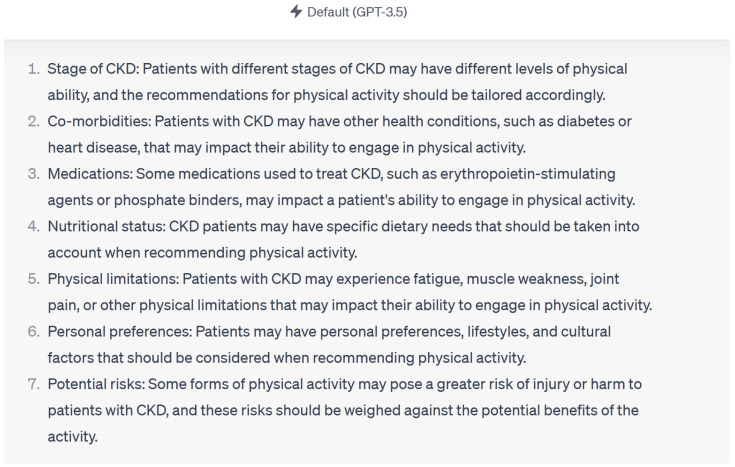
Example of response by Chat GPT 3.5 using KDIGO guidelines.

Overall, the response to queries composed of original questions, paraphrased questions with different interrogative adverbs, paraphrased questions with incomplete sentences, and paraphrased questions with misspelled words was similar. ChatGPT 3.5 (March 2023 version) provided accurate responses to 13 (87%) out of 15 questions across various complexity levels and paraphrasing variations. In two questions, ChatGPT made statements that could have been misleading to patients and healthcare providers. These statements were “medications like erythropoietin stimulating agents and phosphate binders may affect the patient’s ability to engage in physical activity”, (Figure 2) “high protein intake can result in accumulation of metabolites like ammonia in tubular cells”, and “excessive protein intake can activate renin angiotensin aldosterone system”. These statements may be misleading as they are not proven in human studies. Consultation with a nephrologist was encouraged by ChatGPT to individualize care throughout all the questions.

### 3.2. Chat GPT 3.5 (September 2023 Version) for KDIGO Guideline Questions

**Figure 3:**
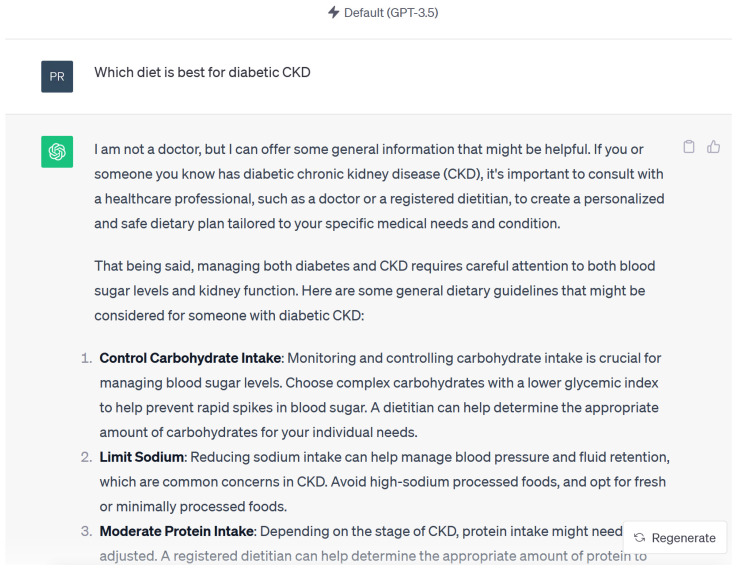
Example of response by Chat GPT 3.5 using KDIGO guidelines.

Responses were accurate and acceptable in 15 out of 15 questions when reviewed by nephrologist. A common statement in ChatGPT 3.5 (September 2023 version) was “I’m not a doctor, but I can offer some general information” (Figure 3) “based on information available up to September 2021, personalized recommendations from healthcare professionals familiar with the patient’s medical history are crucial”. ChatGPT was unable to provide accurate or medically accepted references even when specifically asked requested [25]. When asked for these references, the ChatGPT would state “I apologize, but I cannot provide specific references or cite recent studies as my knowledge is based on information available up to September 2021, and I do not have direct access to external sources or databases to retrieve specific references. However, I can provide you with some general information…”

### 3.3. Chat GPT 4 (September 2023 Version) for KDIGO Guideline Questions

**Figure 4:**
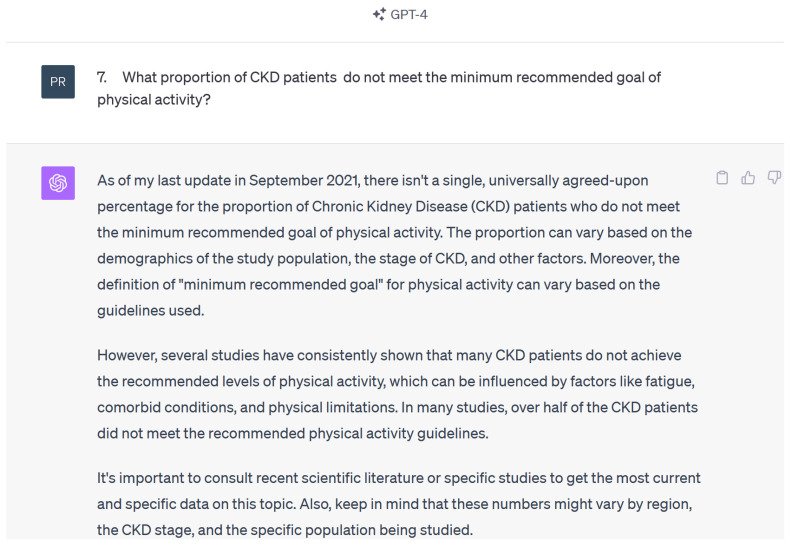
Example of response by ChatGPT 4 using KDIGO questions.

Responses were accurate and acceptable in 15 out of 15 questions when reviewed by the nephrologists. Certain answer responses were presented with more bullet points and thus were lengthier when compared to Chat GPT 3.5 version. ChatGPT 4 was able to provide references when requested, but the reference would not always directly answer the posed question or be applicable. Chat GPT 4 also mentioned a similar date of training to ChatGPT 3.5 “As of my last training data, which covers literature up to 2021….” (Figure 4).

### 3.4. Bard AI (September 2023 Version) for KDIGO Guideline Questions

**Figure 5:**
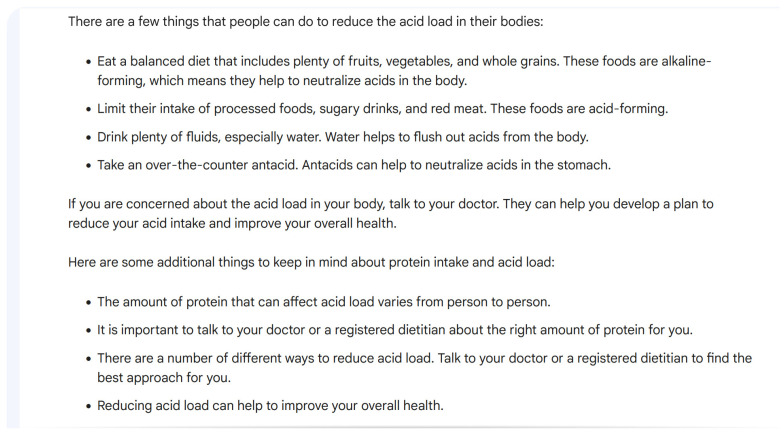
Example of response by Bard AI using KDIGO questions.

Overall, the responses were accurate in 13 (87%) out of 15 questions. Misleading statements included “Moderate-intensity aerobic activity means that you can talk but not sing during the activity [26]”. For the question on how to reduce acid load in the body, Bard AI stated, “Take an over-the-counter antacid. Antacids can help to neutralize acids in the stomach.”, as shown in Figure 5. This is an inaccurate statement in the context of acid–base balance in kidney disease. Specific articles were also not mentioned, but Bard AI stated “Kidney International”, “Nephrology, dialysis, transplantation”, “Nature Reviews”, and “Nephrology” as sources of information for certain questions. Non-peer-reviewed references included “Houston Medical times” and “Atlanta Dunia” for one question.

### 3.5. ChatGPT 3.5 (November 2023 Version) for KDOQI Guideline Questions

**Figure 6:**
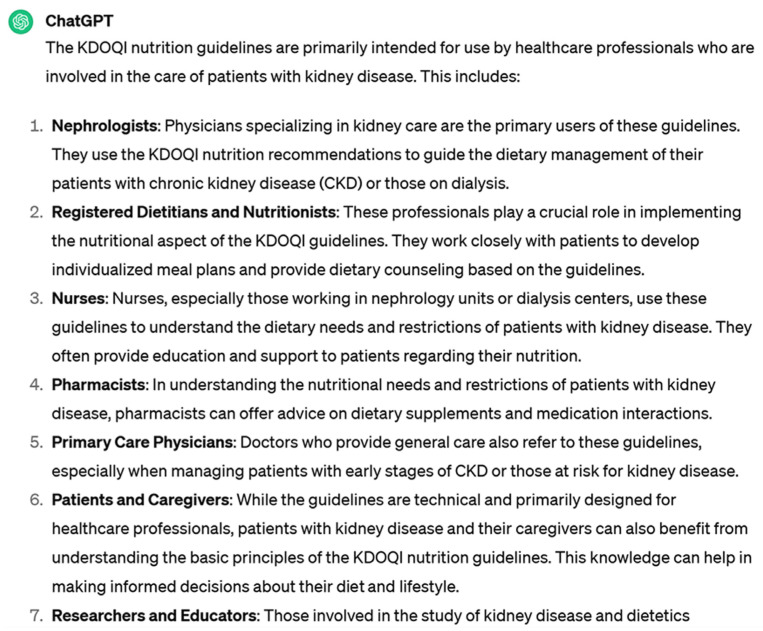
Example of response by ChatGPT 3.5 using KDOQI guidelines.

Overall, the responses were accurate in 20 out of 20 questions. For KDOQI guidelines, ChatGPT stated, “While the guidelines are technical and primarily designed for healthcare professionals, patients with kidney disease and their caregivers can also benefit from understanding the basic principles of the KDOQI nutrition guidelines”, as shown in Figure 6. Generic information in easily understandable layman terms was provided. Consultation with a nephrologist was recommended. Protein intake recommendations differed slightly from KDOQI guidelines.

### 3.6. ChatGPT 4 (November 2023 Version) for KDOQI Guideline Questions

**Figure 7:**
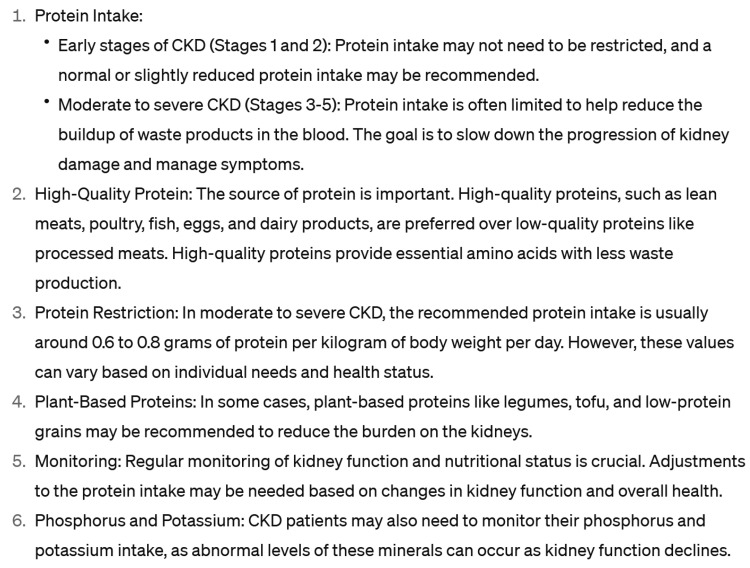
Example of response by ChatGPT 4 using KDOQI guidelines.

Overall, there were no misleading responses in 20 out of 20 questions. Notably, there was minimal difference in the quality of information between ChatGPT 3.5 and the subscription-based ChatGPT 4, as shown in Figure 7. Easily understandable layman terms were used, and consultation with a nephrologist was encouraged. There was a discrepancy in protein intake recommendations in the responses compared to KDOQI guidelines.

### 3.7. Bard AI (November 2023 Version) for KDOQI Guideline Questions

Responses were acceptable in all 20 out of 20 questions, but protein intake recommendations differed slightly from KDOQI guidelines. In fact, Bard AI gave the most accurate and explicit answers along with pictorial representation, as judged by the nephrologists. Consultation with nephrologists was encouraged by Bard AI as well.

### 3.8. Bing AI (November 2023 Version) for KDOQI Guideline Questions

Overall, there were no misleading responses in 20 out of 20 questions. However, the protein intake recommendation was discrepant from KDOQI guidelines. Bing AI provided the shortest answers without much elaboration. At times, the answer response was simply “YES”.

Interestingly, we did not observe significant differences in the content of information provided across the various question formulations for each topic. This consistency suggests a degree of reliability in the AI’s responses, at least within a short timeframe and for closely related query formats.

## 4. Readability

The average Flesch–Kincaid Grade Level for the questions was 10.2 ± 1.8. For the AI-generated responses, the average grade levels were as follows: ChatGPT 3.5 (11.3 ± 2.1), ChatGPT 4.0 (11.1 ± 1.9), Bing AI (11.5 ± 2.3), and Bard AI (11.2 ± 2.0). One-way ANOVA revealed no statistically significant differences between the AI models (F(3,76) = 0.24, p = 0.87). These scores suggest that to fully comprehend the content from all AI models, a similar level of education is required—approximately equivalent to a high-school junior level.

## 5. Discussion

The use of chatbots, especially in the field of medicine, has gained substantial attention since their introduction in 2022. These chatbots have shown significant promise in providing timely, widespread, and individualized lifestyle and nutrition education to CKD patients [27]. Personalized education is known to have a positive impact on healthcare outcomes, and chatbots offer a promising avenue for improving patient engagement and augmenting the efforts of the nephrology healthcare field [5].

The findings of this study provide a comprehensive assessment of the performance of various AI chatbot models in the context of delivering personalized patient education for CKD. Notably, ChatGPT 3.5, (March, September, and November 2023) versions, ChatGPT 4 (September and November 2023), Bard AI (September and November 2023) and Bing AI (November 2023) were evaluated for their effectiveness in providing information on lifestyle modification and nutrition management for CKD patients. In terms of accuracy and consistency, ChatGPT 3.5 exhibited good performance, consistently offering accurate responses to a range of questions irrespective of the question format and complexity. However, there were occasional occurrences (2 out of 15 questions) of misleading statements (like in 2 out 15 questions in March version) within its responses, which may lead to confusion or harm among patients and healthcare professionals. Importantly, ChatGPT 3.5 recommended consulting with a nephrologist, emphasizing the importance of personalized care. The limitations of ChatGPT 3.5 (September 2023 version) included outdated data, as it frequently noted that its last update was September 2021. Additionally, the inability to provide specific references or cite recent studies also limits its credibility and the application of the information shared. ChatGPT 3.5 (November 2023 version) was used to evaluate questions using KDOQI guidelines, and overall responses were generic but accurate and did recommend consulting with nephrologist.

ChatGPT 4, while maintaining the essence of ChatGPT 3.5, presented responses in a more detailed manner with an increased use of bullet points. It exhibited an improvement in reference citation compared to 3.5, although the references to prompts were not consistently relevant. Furthermore, both versions of ChatGPT mentioned the same training date, potentially causing users to question the accuracy of the information. Overall, there was minimal difference in the quality of information between ChatGPT 3.5 and the subscription-based ChatGPT 4.

Regarding Bard AI’s performance, it generally provided accurate responses but not without significant errors. The September 2023 version featured misleading statements in 2 out of 15 questions that could confuse users. Furthermore, its reliance on sources like “Houston Medical Times” and “Atlanta Dunia” may not be deemed best peer-reviewed sources by medical experts. Bard AI (November 2023 version) appeared more accurate and reliable for questions from KDOQI guidelines, as it did not give any misleading information and provided more pictorial representation for which would be easily understood by patients.

Bing AI (November 2023 version) was used only to evaluate questions from KDOQI guidelines, and the answers provided were usually very short without much detail.

This study underscores the significant potential of AI chatbots in delivering personalized patient education for CKD. Evaluations of questions relating to patient education at different time periods noted a pattern of improvement in the responses. Two popular nutrition guidelines in CKD (KDIGO and KDOQI) were used to frame questions to evaluate the depth of accuracy of Chatbots. Their use of layman terms, quick response times, and multilingual support enhances their effectiveness as tools for disseminating medical information and engaging patients in meaningful conversations.

The readability analysis revealed important insights into the accessibility of the AI-generated patient education content. While there were no significant differences in readability levels among the AI models, the overall grade level (approximately 11th grade) suggests that there is room for improvement across all platforms to ensure that the content is comprehensible to individuals with lower educational attainment. Importantly, previous studies have shown that GPT models can effectively reduce readability grade levels when given specific instructions to do so [28,29]. This suggests that despite our current outputs being at approximately 11th grade level, more specific additional prompt instructions in future applications may further improve readability to lower grade levels. The future development of these AI chatbots for patient education should therefore prioritize incorporating explicit instructions for simplified language into the prompts. This approach could potentially enhance the accessibility and effectiveness of AI-generated content across diverse patient populations with varying levels of health literacy.

The introduction of AI chatbots in nephrology and their evident limitations underscore a pressing need for ongoing advancements and synergies. The focus of upcoming research and efforts should include mitigating misinformation specific to CKD management; providing relevant, peer-reviewed, and high-quality medical references; and ensuring that the chatbots’ content remains abreast of contemporary medical practice as reflected by national societal guidelines. These efforts necessitate not only technical enhancements but the close collaboration of nephrologists and AI specialists. By assimilating the expertise of nephrologists in the chatbot refinement processes, patient accessibility to life-changing information with precision can be achieved. It is imperative to position these chatbots as adjunctive tools that complement, but never substitute, the nuanced judgment and experience of a nephrologist. In pursuit of enhancing trust and efficacy in CKD patient education, fostering alliances between AI companies and esteemed nephrology institutions and societies may prove invaluable.

This study, while illuminating the realm of AI application for CKD patient education, bears several limitations. First, the set of 15 questions from KDIGO and 20 questions from the KDOQI guidelines may not encompass the entirety of lifestyle and nutrition complexities faced by CKD patients. This limited scope might restrict the breadth of evaluation for the Chatbot models. Second, while various question formats (including paraphrasing and misspellings) were utilized to mimic patient inquiries, real-world patient inquiries may be more varied and multifaceted. Moreover, the reliance solely on nephrologists’ assessments of chatbot accuracy without the inclusion of patients’ perceptions or feedback might provide a skewed understanding of chatbot efficiency in patient interactions. In addition, only four AI models were evaluated, leaving out potential alternative platforms that might also be beneficial in this context. While our study utilized various question formats to simulate patient inquiries, including paraphrasing and deliberate misspellings, we acknowledge that real-world patient questions may be even more diverse and nuanced. A key limitation of our approach is the absence of direct patient input in formulating these questions. Future studies would benefit significantly from collecting and incorporating questions directly from CKD patients, as this would provide a more authentic representation of the information needs and communication styles of the target population. By implementing these strategies, future studies can more accurately assess the real-world effectiveness of AI chatbots in addressing patient concerns and providing personalized education. This patient-centered approach would yield more robust and clinically relevant results, ultimately improving the practical applicability of AI-driven patient education tools in nephrology care. Additionally, a comparative analysis involving a broader range of AI platforms in nephrology education would be insightful, ensuring a comprehensive evaluation of available tools for patient-centric education. Lastly, with AI being a rapidly changing field, more studies using newer AI models over extended periods and with exact repetitions of questions will be able to delineate the fast progress in AI over time. This continuous evaluation is crucial to keep pace with technological advancements and ensure that findings remain relevant to current capabilities. Our methodology can be adapted and applied to evaluate newer AI models as they emerge, ensuring the ongoing assessment of their suitability for patient education in chronic kidney disease management.

While our study focused on patient education in CKD management, we acknowledge that a more comprehensive evaluation of AI in medicine should encompass its diagnostic and therapeutic capabilities, especially in challenging cases where guidelines may not provide definitive recommendations. This approach would indeed offer a more rigorous assessment of AI’s potential in clinical decision-making. We recognize that expanding our research to include complex clinical scenarios would provide valuable insights into AI’s capacity to assist in diagnostic reasoning and treatment planning, particularly in areas where evidence-based guidelines are limited or conflicting. For future research in this direction, we propose developing a set of complex case scenarios in nephrology, focusing on diagnostic dilemmas and treatment decisions where current guidelines offer limited direction. These cases would be presented to various AI models to collect their diagnostic reasoning and treatment recommendations. A panel of expert nephrologists would then evaluate the AI responses, judging them on criteria such as clinical reasoning, evidence basis, and potential patient outcomes. Comparing AI performance against that of nephrology trainees or general practitioners could gauge its potential as a clinical support tool. This approach would not only assess AI’s current capabilities but also highlight areas for improvement in AI models and potentially reveal novel approaches to complex clinical problems. Moreover, it could inform the development of more sophisticated, clinically oriented AI tools for nephrology practice, ultimately enhancing patient care and medical education in the field. The future development of AI for patient education should strike a balance between adhering to established medical guidelines and leveraging AI’s capability to provide personalized, context-specific information. This could involve training AI models on a curated dataset of high-quality medical information, including but not limited to guidelines, and developing sophisticated algorithms to synthesize this information in response to patient queries. Such an approach would enhance AI’s ability to handle complex cases while maintaining a foundation in evidence-based practice.

## 6. Conclusions

While AI chatbots like ChatGPT, Bing AI and Bard AI hold promise for patient education in the context of CKD, they currently have significant limitations. This study highlights the need for the ongoing refinement of these AI models to reduce misleading statements, improve reference quality, and provide accurate updated knowledge. Using vetted, high-quality, trustworthy medical sources like national and international guidelines would avoid the presentation of incorrect information and keep chatbot content accurate, in line with current medical practice. Ultimately, a collaborative approach between healthcare experts, patients, and AI developers is key to harnessing the potential of AI in CKD care while ensuring accuracy and reliability.

## Data Availability

The data underlying this article will be shared on reasonable request to the corresponding author.

## Supplementary Materials

The following supporting information can be downloaded at: https://www.mdpi.com/article/10.3390/diseases12080185/s1, Table S1: Original Questions from KDIGO guidelines; Table S2: Original questions from KDOQI guidelines.
