# Supplementary material for: AI-Driven Patient Education in Chronic Kidney Disease: Evaluating Chatbot Responses against Clinical Guidelines
Source: Diseases. 2024 Aug 16;12(8):185. doi: 10.3390/diseases12080185 (PMC11353724; doi:10.3390/diseases12080185)
Supplement: Supplementary file 1 [file diseases-12-00185-s001.zip › diseases-3088286-supplementary.pdf]

Supplementary table 1

Original Questions from KDIGO guidelines

1.What is the recommended exercise duration for CKD patients

2. What is the recommended BMI for CKD patients

3. How does exercise affect cardiovascular disease?

4. What is the recommended moderate intensity physical activity for diabetic CKD?

5. Which factors should be considered while recommending physical activity to CKD patients?

6. Is sedentary behavior good for CKD patients?

7. What proportion of CKD patients do not meet the minimum recommended goal of physical activity?

8. Does smoking increase risk of kidney failure?

9. Does smoking contribute to worsening/progression of CKD

10. Does weight loss help decrease proteinuria

11. What is the recommended protein intake in CKD stage 4,5 patients not on dialysis.

12. What is the recommended protein intake in dialysis patients

13. Which diet is best for diabetic CKD

14. Does high protein intake affect acid load in body

15. What is the pathophysiology of high protein intake causing worsening kidney function?

Paraphrase version with different interrogative adverbs from KDIGO guidelines

1. How long should CKD patients exercise for, according to recommendations?

2. According to recommendations, what should be the BMI of CKD patients?

3. In what way does exercise impact cardiovascular disease?

4. What is the recommended moderate intensity physical activity for CKD patients with diabetes?

5. What factors should be taken into account when recommending physical activity to CKD patients?

6. Is being sedentary recommended for CKD patients?

7. What proportion of CKD patients fail to meet the minimum recommended physical activity goal?

8. Is there an increased risk of kidney failure associated with smoking?

9. Does smoking contribute to the progression/worsening of CKD?

10. Can weight loss aid in reducing proteinuria?

11. According to recommendations, what is the recommended protein intake for stage 4 and 5 CKD patients not on dialysis?

12. According to recommendations, what is the recommended protein intake for dialysis patients?

13. What is the best diet for CKD patients with diabetes?

14. Does high protein intake affect the body's acid load?

15. What is the underlying pathophysiology of high protein intake causing a decline in kidney function?

Paraphrase version with different interrogative adverbs; additional remove verbs and prepositions from KDIGO guidelines

1. What is the recommended exercise duration for CKD patients?

2. What should be the BMI of CKD patients as per recommendations?

3. How does exercise impact cardiovascular disease?

4. What moderate intensity physical activity is recommended for diabetic CKD patients?

5. Which factors need to be considered while recommending physical activity to CKD patients?

6. Is sedentary behavior good for CKD patients?

7. What percentage of CKD patients fail to meet the minimum recommended physical activity goal?

8. Does smoking increase the risk of kidney failure?

9. Does smoking contribute to CKD progression/worsening?

10. Can proteinuria be reduced with weight loss?

11. What is the recommended protein intake for stage 4 and 5 CKD patients not on dialysis, as per guidelines?

12. What is the recommended protein intake for dialysis patients, according to recommendations?

13. Which diet is considered best for CKD patients with diabetes?

14. Does high protein intake affect the body's acid load?

15. What is the underlying pathophysiology of high protein intake leading to worsening of kidney function?

Paraphrase version with different interrogative adverbs:additional remove verbs and prepositions with misspelled words from KDIGO guidelines

1. What is the recommended excersise duraton for CKD patiens?

2. What should be the BMI of CKD patiens as per recomendations?

3. How does excersize impact cardivascular disease?

4. What moderte intensity physical activity is recomended for diabbetic CKD patiens?

5. Which factors need to be considdred while recomendig physical activity to CKD patiens?

6. Is sedentary behavir good for CKD patiens?

7. What persentage of CKD patiens fail to meet the minimum recommended physical activty goal?

8. Does smoking increse the risk of kidney failur?

9. Does smoking contribut to CKD progession/worsenig?

10. Can protinuria be reduced with wieght loss?

11. What is the recommended protin intake for stage 4 and 5 CKD patiens not on dialisis, as per guidlines?

12. What is the recomended protien intake for dialisis patiens, according to recommendations?

13. Wich diet is considered best for CKD patiens with diabtes?

14. Dose high protin intake affect the body's acid lod?

15. What is the underliyng pathofisiology of high protin intake leading to worsenig of kidney function?

Supplementary Table 2

Original questions from KDOQI guidelines

1. What are the KDOQI guidelines?

2. Who should use the KDOQI nutrition guidelines?

3. What areas do the KDOQI nutrition guidelines focus on?

4. What is medical nutrition therapy (MNT) in the context of CKD?

5. Why is protein intake important for patients with CKD?

6. How does CKD affect energy intake needs?

7. What role do nutritional supplements play in CKD?

8. Why are micronutrients important for patients with CKD?

9. How do electrolytes relate to CKD management?

10. Should patients with CKD make lifestyle changes?

11. Can diet affect the progression of CKD?

12. Are there specific dietary protein recommendations for CKD patients?

13. How should CKD patients manage their salt intake?

14. Why is potassium management important in CKD?

15. What types of food should CKD patients be cautious about?

16. Is physical activity recommended for CKD patients?

17. Should CKD patients take vitamin and mineral supplements?

18. How does CKD affect calcium and bone health?

19. Can lifestyle changes reduce the risk of cardiovascular disease in CKD patients?

20. How important is fluid management for CKD patients?
